# Resilience and sense of coherence in first year medical students - a cross-sectional study

**DOI:** 10.1186/s12909-021-02571-5

**Published:** 2021-03-04

**Authors:** Lena Luibl, Julia Traversari, Friedrich Paulsen, Michael Scholz, Pascal Burger

**Affiliations:** 1 Institute of Functional and Clinical Anatomy, Friedrich-Alexander-University Erlangen-Nürnberg, Universitätsstr. 19, 91054 Erlangen, Germany; 2 Institute of Anatomy, University of Zurich, Winterthurerstrasse 190, 8057 Zürich, Switzerland; 3 Department of Psychiatry, Psychiatric University Hospital, Lenggstrasse 31, 8008 Zurich, Switzerland

**Keywords:** Mental health status, Undergraduate medical students, Salutogenesis, Resilience, Sense of coherence, Medical education

## Abstract

**Background:**

A broad range of studies show that medical students often have a significantly deteriorated mental health status. Although starting medical school with values comparable to the population average, only a few semesters later, medical undergraduates show increased rates of psychological risk states and some manifest mental illnesses, such as burnout and depression. In our survey we intentionally assessed mental health parameters from a salutogenetic, i.e. resource-oriented point of view.

**Methods:**

We examined first-year medical students in a cross-sectional study and assessed sense of coherence (SOC) and resilience as parameters from the salutogenesis model by Antonovsky in a structured way using validated, self-administered questionnaires. In total, we examined 236 students of human medicine, dentistry and molecular medicine at the Friedrich-Alexander-University Erlangen-Nürnberg (FAU).

**Results:**

Our analyses showed significantly higher values of resilience among male students compared to female students (*p* < 0.01). In contrast, even though a significant correlation between resilience and SOC was observed, only a non-significantly lower value of SOC was found in female students. Compared to the reference sample our medical students in their first year of study showed significantly lower values for resilience (*p* < 0.01) and SOC (*p* < 0.01).

**Conclusion:**

Resilience and SOC are known to correlate with psychological stress (burnout parameters) and depression. In order to keep protective factors like SOC and resilience in medical students at a good and healthy level we see the necessity to address that problem proactively and educationally. Integrating training focused on the preservation of the students´ own mental health into the medical curriculum from the beginning of university courses, and throughout the whole medical study course, is essential and should be an obligatory training goal. Based on our study results, we also deem it necessary to think about ways to adapt the measures for the gender-specific needs of our students, e.g. dependent on their biological gender.

## Background

Physicians must be able to care for their patients in a holistic way. In other words, not only by treating cases of acute or chronic illness, but also by implementing strategies to prevent mental and physical disorders. However, compared to the general population, physicians themselves show a massively increased prevalence of mental disorders such as depression, anxiety disorders or substance use disorders [1–4]. Many studies [5–7] have successfully demonstrated that undergraduate physicians already show a very high frequency of risk factors for mental health problems [1, 8, 9] such as burnout, and some manifest mental disorders [10–16]. This is particularly noteworthy, as the future physicians seem to begin their studies with increased levels of disorders such as depression and anxiety disorders compared to the general population [1, 17–19].

To our knowledge, no obligatory courses, activities or learning goals to prevent this problem are integrated in the medical curriculum to this day. Nevertheless, over the last years, the awareness on the topic of increased levels of psychological disorders among medical students has risen. Thus, extracurricular programs on the improvement of mental health [20–25] and student interest groups, like the Lachschaft, an initiative by medical students in Erlangen for the promotion of mental health, have been established.

**Fig. 1:**
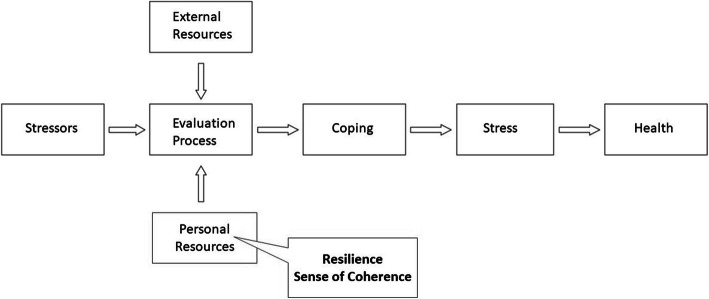
Adapted salutogenesis model to show the role of SOC and resilience

Not only the presence of mental disorders (or potentially already existing damage), but particularly the preconditions and mental health resources that students possess should be assessed and ameliorated. In this study we refer to Aaron Antonovsky’s salutogenesis model, which examines the factors that benefit health and focuses on personal resources like resilience and sense of coherence. In this model (Fig. 1) the development of disorders and preservation of mental health depends on the individual perception and assessment of stressors and the reaction to the latter. Resilience represents the individual ability to deal with stressors and integrate them into daily life without persistent psychological damage. As a possible protective mechanism in actively preventing mental disorders, resilience encompasses behaviors and thoughts which can be learned and developed, such offering much curricular promise [26]. Sense of coherence (SOC) describes a global orientation that expresses the extent to which one has a pervasive, enduring though dynamic feeling of confidence. It states that (i) the stimuli that arise from the inner and outer environment throughout life are structured, predictable and explainable, (ii) the individual has the resources to meet the demands these stimuli make, and (iii) these demands are challenges that are worth engaging with and explaining. Thus, the SOC can be interpreted as a mean to measure one’s attitude towards life. Antonovsky describes that a person with a strong SOC can perceive the uniqueness of each situation in which he or she is involved and can thus be flexible [27]. The SOC comprises of 3 components, comprehensibility, manageability and meaningfulness. According to Antonovsky, meaningfulness is the most important component and is therefore most strongly represented. Regarding these 3 factors, the possible importance of the SOC for medical training and teaching can be deduced. For achieving the goal to become a medical expert, students need learn to understand, classify and manage situations for their patients´ best outcome. The constellation of the SOC’s components results in the overall SOC and consequently describes the stability of an individual’s psychological constitution. SOC and its components alike are closely linked to external and social resources. The aim of our study was to examine the mental health of medical students using two salutogenic factors, i.e. the parameters of resilience and SOC, which were specifically chosen as we consider them to be of particular importance in a physician’s working environment and behavior due to their strong correlation with mental health [27–30]. These results could demonstrate a need and the opportunity to study and adapt didactical and interventional approaches for the improvement of mental health in medical students by using these specific parameters. Considering that a majority of medical students is female [31] with constantly rising numbers of women starting to study medicine and regarding the gender-related discrepancy in psychological health issues [32], we were especially interested in whether there is a difference with regard to the salutogenetic factors between genders. This additional dimension could allow the development of more tailored course offers aiming at the prevention of mental health risks and keeping our students functional.

## Methods

We conducted our study surveying first year medical students from all 3 medical study curricula (human medicine, dentistry and molecular medicine) offered in Erlangen.

### Measuring instruments

The RS-13 is a validated, self-administered survey for the measurement of mental resilience [24, 33] and is an abridged version of the RS-25 which covers several subscales: purposeful life, perseverance, equanimity, self-reliance and existential loneliness [23]. In contrast, the RS-13 measures resilience as a one-dimensional global value. By summing up the values, the test persons are divided into “low, moderate and high resilience”. A score of up to 66 points is considered to signify low, up to 72 points moderate and 73 points and above high resilience.

The SOC L-9 is the validated short form (“Leipziger Kurzfassung”) of the standard survey for measuring SOC. Values for the German general population for the SOC-scale were established in a survey including almost 2000 participants, several short versions, including the SOC-L9, were tested to be valid and reliable for sufficiently estimating the SOC in German participants [34]. The SOC L-9, comprises 9 items, which achieve a high measuring accuracy (Cronbach’s Alpha = 0.87) [35, 36]. Particularly, it tests for comprehensibility, manageability and meaningfulness giving these factors different weight. According to Antonovsky, meaningfulness is the most important component and is therefore most strongly represented. The evaluation is carried out by summation, a higher value indicates a stronger expression of the SOC.

Statistical analysis was conducted using the statistical software R [37]. Beside descriptive statistics we performed tests for normal distribution (Kolmogorov-Smirnoff-test) and tested – as there was a normal distribution – for differences by using t-tests. Furthermore, we conducted a one-way-ANOVA and a chi-square (χ^2^) test estimation the association of factors with our results and performed a correlation analysis.

This study is a subsegment of the ESTRELLAS surveys on medical students, approved by a positive vote of the Ethics Committee of the Friedrich-Alexander-University Erlangen-Nürnberg (FAU).

## Results

During the winter semester 2017, a total of 236 medical students took part in the survey. There were no drop-outs within the groups and all participating students handed in their questionnaires filled out completely. The distribution of the study participants according to the study programs and biological gender can be seen in Table 1. In each of the medical courses of studies, an unequal gender distribution in favor of female students was observed. This was most evident in students of molecular medicine, with more than 80% female participants.

**Table 1:**
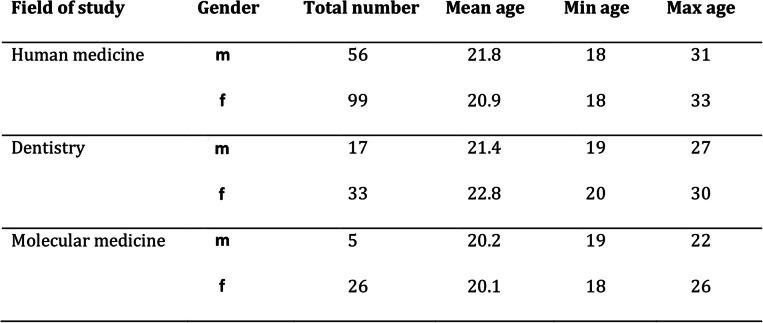
Probands from medical courses of studies

### Resilience

**Fig. 2:**
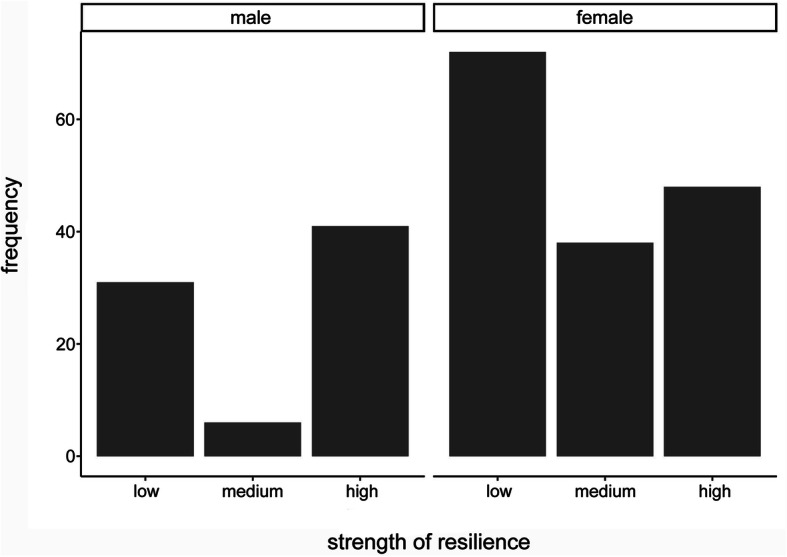
Frequency of resilience expressions according to RS-13 in the gender groups

**Fig. 3:**
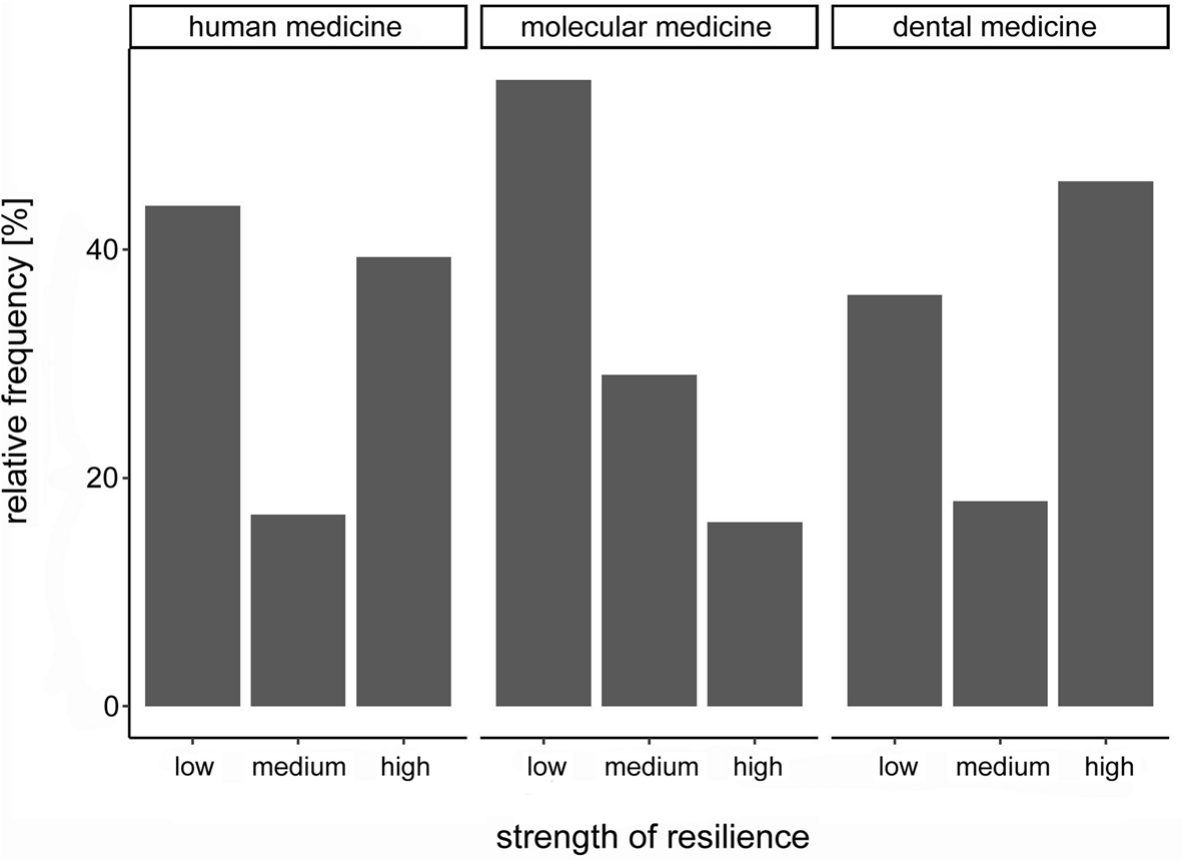
Frequency of resilience expressions according to RS-13 in the sub collectives

The descriptive statistics of the resilience values according to RS-13, both overall and in the subgroups, divided by gender, are shown in Table 2. Accordingly, subjects whose aggregated resilience value ranks between 13 and 66 have low, between 67 and 72 moderate and between 73 and 91 high resilience. A test for normal distribution of the original data was negative, but due to the central limit theorem, the test statistic based on *n* = 236 follows a t-distribution (asymptotic t-test). Differences in the mean resilience scores between gender groups were analyzed by carrying out a t-test and were considered significant at *p* ≤ 0.05 (difference = 3.56, CI(0.95) = [0.32; 6.81]). The differences between male and female students were statistically significant in the whole study group (*p* < 0.01). Furthermore, we conducted a chi-square (χ^2^) test to identify associations between gender and those resilience levels. In our group of students, a distinctly greater amount of highly resilient individuals was present in the male student group (statistic = 14.72; df = 2; *p* < 0.01) (Fig. 2). The distribution of the resilience levels within the respective student subgroups (human medicine, dental medicine, molecular medicine) is presented in Fig. 3. There were no statistically significant differences detectable between those groups (statistic = 8.51; df = 4; *p* = 0.07). Overall, the mean value for resilience in the general population is 70 points (range: 13–91, SD = 12) [33]. The comparison between our medical students and the reference sample by means of a t-test showed that the medical students have significantly lower average resilience scores (difference = − 2.44, CI(0.95) = [− 3.96; − 0.92]; *p* < 0.01).

**Table 2:**
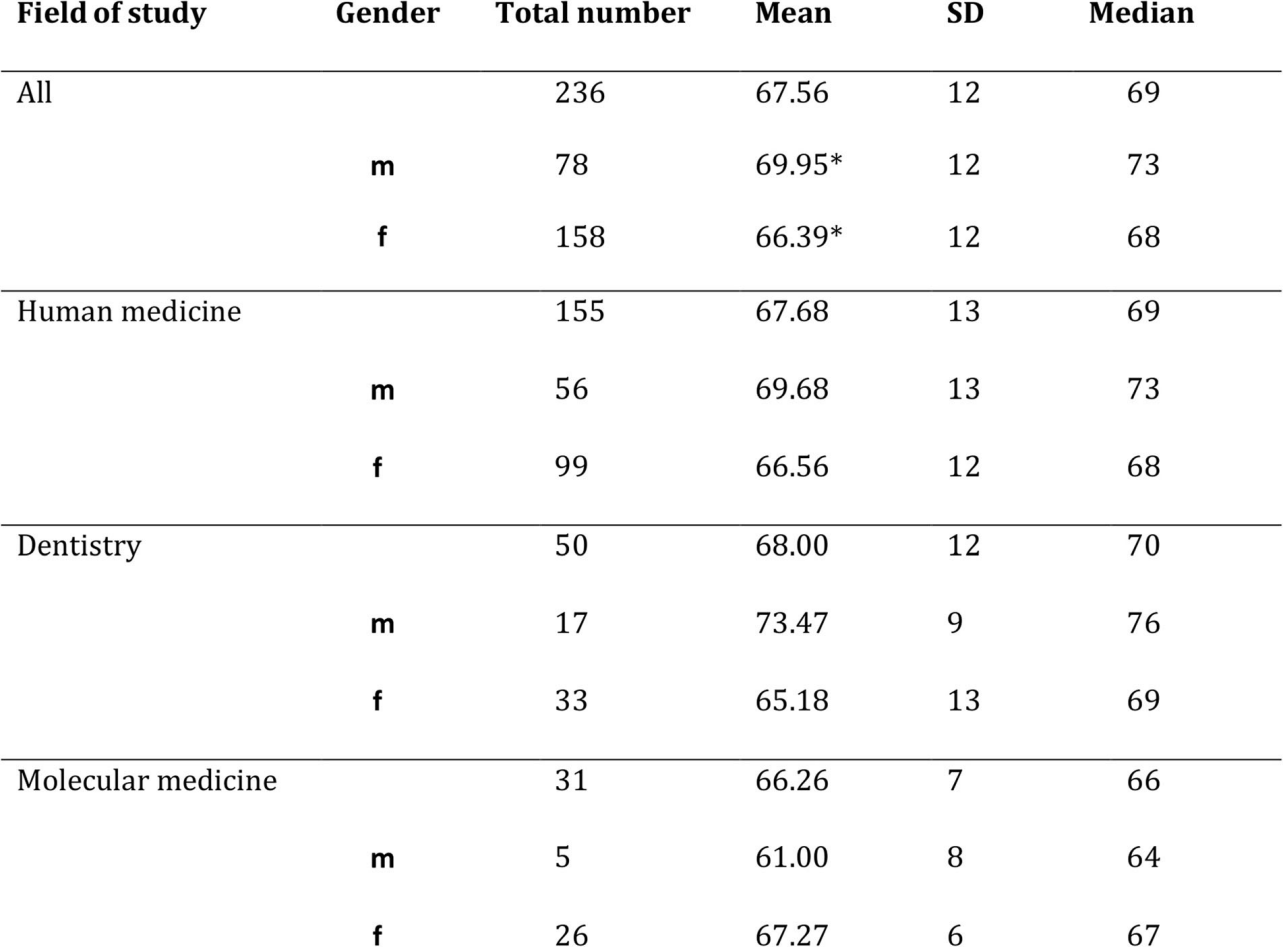
Values given by test participants for resilience, subdivided according to fields of study and gender. *= significant difference with *p*<0.01.

### Sense of coherence

**Fig. 4:**
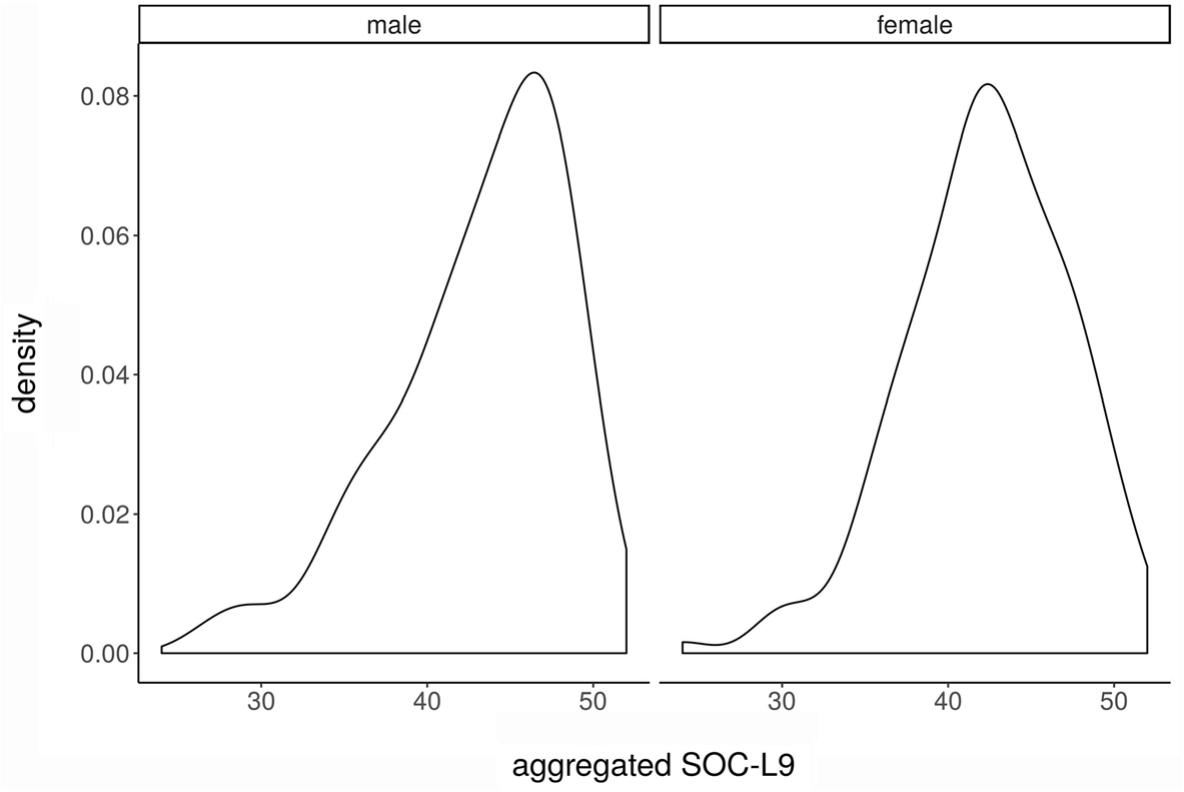
Kernel density estimates of SOC L9 values in the gender groups

**Fig. 5:**
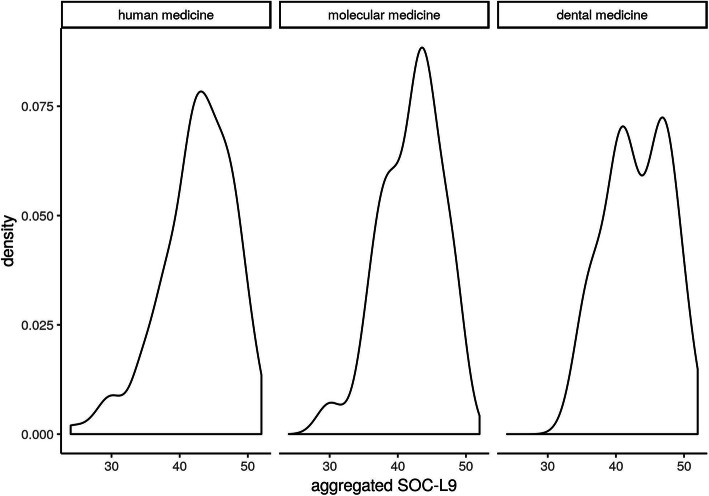
Core density estimates of SOC L9 values in the subcollectives

Table 3 presents total number, mean, median and SD of the SOC-L9 scores, overall and in subgroups, broken down by gender. In order to test for differences in the mean of SOC, an asymptotic t-test was performed. A slightly higher mean SOC value for male participants compared to female participants (see Fig. 4) was observed. However, in contrast to resilience, there was no statistically significant difference (difference = 0.87, CI(0.95) = [− 0.56;2.30]; *p* = 0.23) detectable between male and female subjects. The analysis of our subgroups within the study program (human medicine, dental medicine, molecular medicine) can be seen in Fig. 5. To test for differences in the mean of the groups, we conducted an anova-test based on a regression of the SOC L-9 values on study program dummies. Here as well there was no statistically significant difference in the absolute SOC L-9 values (*p* = 0.66). Concerning the SOC, our first-year medical students also had significantly lower values compared to the reference sample (mean value for men = 50.68, SD = ±7.77; mean value for women = 49.68, SD = ±7.78 [38]). The results of a t-test comparing all medical students to the reference sample - both overall and categorized by gender - indicate that our group of medical students showed a significantly lower mean value of SOC (medical students: overall: mean = 42.57; CI(0.95) = [41.92; 43.22], *p* < 0.01; male medical students: mean = 43.16; CI(0.95) = [41.95; 44.36], *p* < 0.01; female medical students: mean = 42.29; CI(0.95) = [41.51; 43.07], *p* < 0.01).

**Table 3:**
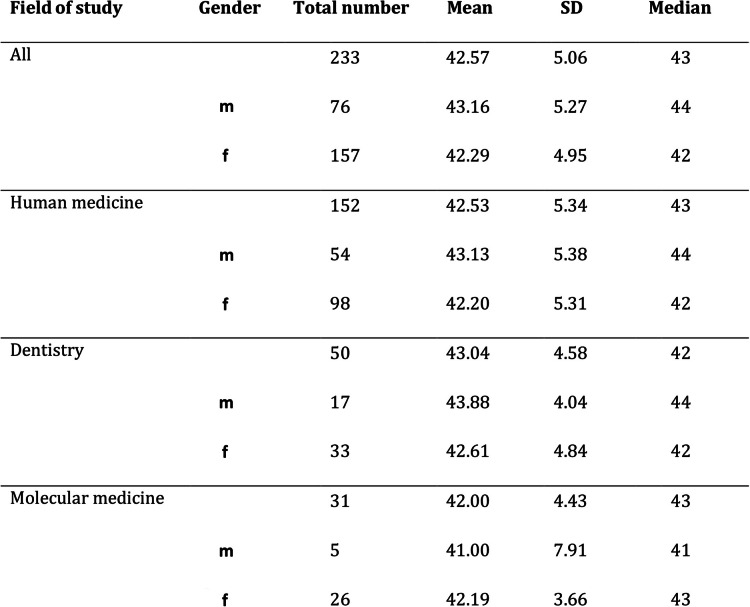
The values given by the participants for SOC, subdivided according to fields of study and gender.

### Relationship between resilience and SOC

**Fig. 6:**
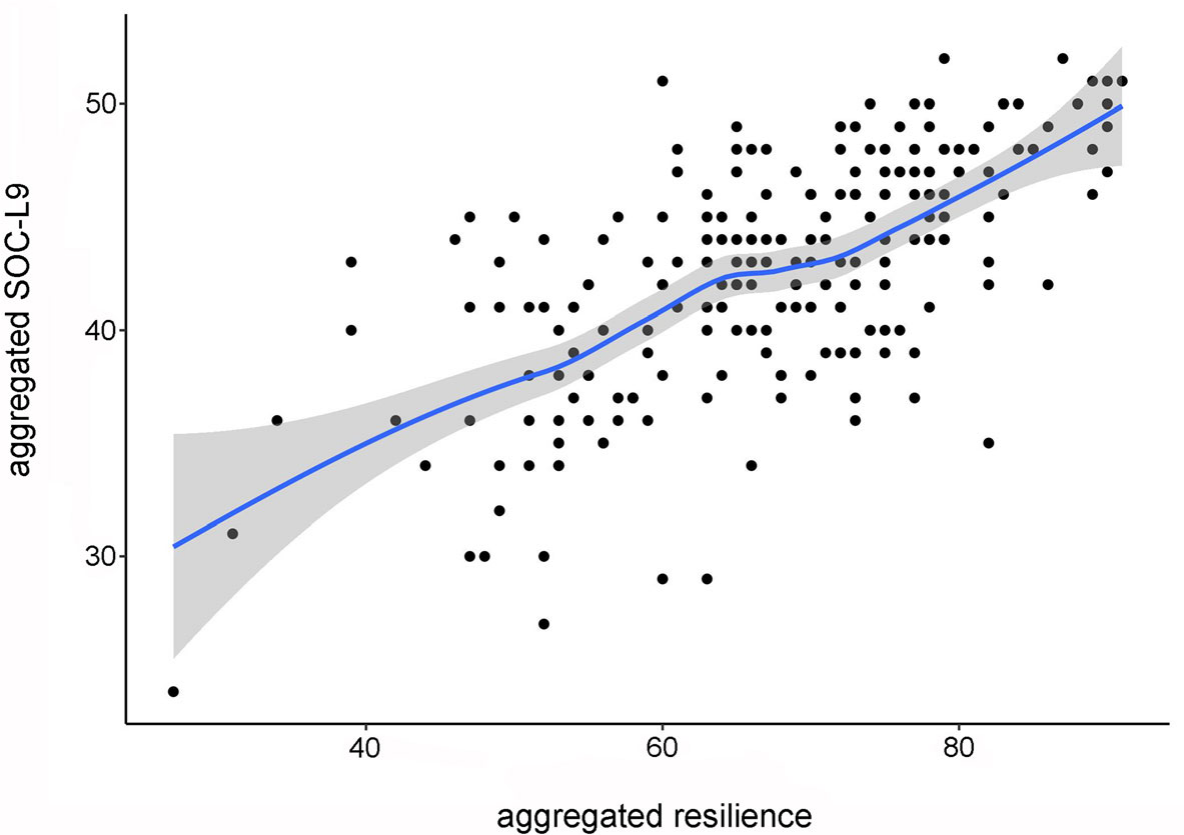
Relationship between resilience and SOC values

Based on the construct of salutogenesis presented in Fig. 1, a connection between the resilience and the SOC seems to be a logical consequence. We performed a correlation analysis between the two parameters (Fig. 6) which revealed a moderate correlation (*R*^2^ = 0.43) between resilience and SOC in the whole study cohort (*N* = 236). In addition, there were no statistically significant differences in the relationship between resilience and SOC with respect to gender.

## Discussion

The results of our studies are in line with recent literature on mental health problems of medical students and physicians [7, 39–43]. In contrast to most studies on medical students which focus on symptoms such as suicidal tendencies or disorders like depression, we examined our collective of future physicians for parameters of mental health using a salutogenetic approach. The factors examined, SOC [27, 28] and resilience [29, 30], are strongly correlated with symptoms of mental disorders such as depression and anxiety disorders. Our data indicate an impairment of those protective factors for mental health after a little more than the first semester of study. We found a large proportion in our group of medical students at that stage who had low to moderate resilience values and distinctly reduced SOC in comparison to the population average. As previously shown in other studies, this circumstance correlates to an increased vulnerability to mental illnesses. This could in turn account for the increased prevalence of mental disorders over the course of medical studies [18, 44, 45]. Due to the character of the parameters we chose, our results also suggest that this development is probably caused by an insufficient adaptation to psychological stress and a lack of coping mechanisms. In an earlier study, we already found lower SOC values in female students [18, 46], and our recent data indicates that the factor resilience follows an even more gender-imbalanced pattern. Although our survey was administered to a relatively small-sized sample group, we were able to find a significant difference between the gender groups with regards to their resilience. Thus, female students not only appear to be at higher risk for psychiatric disorders [47, 48] but also disproportionally lack protective features [32]. This is very relevant for medical students and future physicians for several reasons, since almost two thirds (64.2% in the winter semester 2019/2020) of medical students in Germany are female [49]. A similar trend towards a female majority in the student body can be seen in the US, as reported in 2017 by the Association of American Medical Colleges [31, 42]. Extrapolating our results, we conclude that the majority of medical students find themselves at risk with regards to their relative lack of psychologically protective factors. Physicians have an increased suicide rate compared to all other professions, including the paradoxical finding of female physicians having much higher suicide rates than their male equivalents [50]. We therefore consider gender-specific prevention courses to be all the more important.

Based on our analysis, we conclude that SOC and resilience have a mutual impact on each other. The connection between the two parameters has already been discussed in some studies [51, 52]. This circumstance in turn can possibly influence the development of mental disorders: In case of successful coping with a crisis, the awareness of the problem rises through a higher comprehensibility. A better manageability is perceived and retrospectively a value is recognized in the experienced event, which allows a crisis to be considered meaningful. The more often an individual experience occurs - regaining the original state of mind after a crisis - and the more understandable the situation gets, the more psychologically stable the individual gets. Therefore, it seems plausible that a stronger SOC is connected to a higher resilience – up to a certain extent both factors might be influenced and changed.

Back in 2008, Emma Warnecke wrote: “[...] students are taught about managing the health of others, there is an imperative to provide them with effective, evidence-based ways to manage their own stress.” [53]. We consider it mandatory for medical education and our future physicians to learn evidence-based ways to compensate for their own stress and keep their own mental health intact in a structured manner. Consequently, and in accordance with our approach, medical curricula should be didactically and content-wise orientated towards creating opportunities that allow the participants to grow in line with their challenges. We believe it far more likely for medical students to remain mentally healthy if the course program included resilience-skills as a central learning goal and was adapted to gender-specific needs [2, 18]. Established programmes to improve resilience already exist. For example, “The Road to Resilience” was developed by the American Psychological Association (APA) and “10 ways to build resilience” offers concrete instructions for learning and implementing resilient behaviour [26, 54]. These or similar programs could be adapted for medical students and be integrated as obligatory content at medical schools.

From our own experience and projects at other faculties [23, 25, 55–58], we consider relaxation and mind-body techniques to be feasible and promising options. A firm curricular anchoring of teaching and training these techniques could be achieved by implementing recurring and ultimately self-applied exercises in the curriculum. These measures might result in a higher sensitivity for the future physicians own stress tolerance and the habit of self-administered mental health maintenance [1, 18, 59]. Moreover, to reinforce mental stability, we would recommend e.g. applying the construct of resilience on the didactic blueprint of teaching units. The concept of learning in the context of manageable critical situations could be applied to more than just to emergency medicine, where it is already partly being done. There, for example, training is carried out in a team on a simulation patient. Even if mistakes are being made, in the end the patient survives, the student gets a comprehensible and constructive feedback and repeats the training as long as it is necessary in order to be able to handle the situation sufficiently. With this approach the student has experienced manageability and comprehensibility of the scenario despite an extremely stressful situation [22, 60, 61], dosing the feeling of loss of control and frustration to a manageable level. We recommend to anchor stress management and promotion of resilience as crucial learning objectives in medical education.

## Conclusions

The medical curriculum is known to be a source of severe psychological stress for students and medical students’ average mental health parameters deteriorate significantly over the course of studies. Resilience and SOC make a considerable contribution to the maintenance of mental health and can be seen as resources for university and postgraduate period alike. We therefore consider it essential to integrate preventive courses for mental health and also apply structures that promote salutogenesis in the curriculum to strengthen resistance to mental disorders in medical students. The maintenance of one’s own mental health should be defined as a central learning goal in the curriculum and the courses to achieve it should adapt measures to biological gender.

## Data Availability

The datasets used and analysed in the current study are part of the secured data base of the medical education research unit at the Institute of Functional and Clinical Anatomy.

## Abbreviation

SOC: Sense of coherence
